# Development of microsatellite markers in *Robinsonia* (Asteraceae) an endemic genus of the Juan Fernández Archipelago, Chile

**DOI:** 10.1007/s12686-012-9734-2

**Published:** 2012-08-19

**Authors:** Koji Takayama, Patricio López Sepúlveda, Gudrun Kohl, Johannes Novak, Tod F. Stuessy

**Affiliations:** 1 Department of Systematic and Evolutionary Botany, Biodiversity Center, University of Vienna, Rennweg 14, 1030 Vienna, Austria; 2 Institute for Applied Botany and Pharmacognosy, University of Veterinary Medicine, Veterinärplatz 1, 1210 Vienna, Austria

**Keywords:** Genetic diversity, Oceanic islands, Pyrosequencing, *Robinsonia*

## Abstract

Ten microsatellite markers were developed for *Robinsonia* (Asteraceae), a genus endemic to the Juan Fernández Archipelago, Chile. Polymorphisms of these markers were tested using one population each of *R. evenia*, *R. gayana*, and *R. gracilis*. The number of alleles for these markers ranged from 2 to 17 per locus, and expected heterozygosity ranged from 0 to 0.847 by population. A significant deviation from Hardy–Weinberg equilibrium was observed in zero to two markers in each population, and no significant linkage disequilibrium between markers was detected. The markers reported here would be useful for evolutionary studies and conservation strategies in *Robinsonia*.

Oceanic islands are significant ecosystems for the conservation of global plant diversity due to their small areas and high levels of endemism in comparison with continental regions (Kier et al. 2009). Tiny population sizes and unique characteristics of insular endemic species make them particularly sensitive to anthropogenic disturbances (Frankham 1997). The Juan Fernández Archipelago is located in the Pacific Ocean 667 km west of continental Chile, and it consists of two major islands of different geological ages, Robinson Crusoe Island (4 million years old) and Alejandro Selkirk Island (1–2 million years old) (Stuessy et al. 1984). The flora of the Archipelago contains 132 endemic vascular plants (Marticorena et al. 1998), 74 % of which is regarded as “threatened” based on IUCN criteria (Ricci 2006). Biodiversity assessement in this archipelago, including population genetic study, is a pressing need as is the case with other oceanic islands (Caujape-Castells et al. 2010).

The genus *Robinsonia* DC. (Asteraceae) is endemic to the archipelago, and consists of eight species (Sanders et al. 1987; Danton and Perrier 2006). It should be pointed out that Pelser et al. (2007, 2010) suggested submergence of all species of *Robinsonia* into the large genus *Senecio* in order to maintain strict holophyly of the latter, which would obviate endemic generic status for *Robinsonia*. We do not follow this suggestion, however, as we prefer to recognize *Robinsonia* as generically distinct based on its striking divergence in morphological features, such as a dioecious breeding system and a rosette tree habit. Morphological and ecological divergences occur among *Robinsonia* species, and genetic divergence among them has also been demonstrated with isozyme and ITS markers (Crawford et al. 1992; Sang et al. 1995). In view of the estimated ages of the islands, species of *Robinsonia* have diversified cladogenetically within the past 4 million years (Stuessy et al. 1990). Most of the species in *Robinsonia* are considered to be highly threatened (Ricci 2006). In this present study, we develop microsatellite markers for investigating evolutionary processes within the genus.

We used one individual of *R. masafuerae* Skottsb. for isolation of microsatellites, plus one individual each of *R.* *evenia* Phil., *R. gayana* Decne., *R.* *gracilis* Decne., *R. saxatilis* Danton, and *R.* *thurifera* Decne. for cross-species amplification tests. Polymorphism of microsatellite markers was evaluated in one population each of *R. evenia*, *R.* *gayana*, and *R.* *gracilis*, the most common species on Robinson Crusoe Island.

Total genomic DNA was extracted from leaf tissue by the cetyltrimethylammonium bromide method (Doyle and Doyle 1987) or Qiagen DNeasy 96 Plant Kit (Qiagen, Hilden, Germany). The extracted DNA of *R. masafuerae* was sequenced with one-fourth of the run on 1/8 of a 70 × 75 PicoTiterPlate using a multiplex identifier in 454 Genome Sequencer FLX System (Roche Applied Science, Penzberg, Germany) of LGC Genomics (Berlin, Germany). A total of 32,468 reads with an average length of 425 bp was generated.

The design for primer pairs was conducted with QDD 2.1 (Meglécz et al. 2010) using default settings. A total of 201 unique microsatellite regions contained pure/compound dinucleotide (140 regions) and trinucleotide microsatellite sequences (61 regions) with more than five repetitions, and primer designable flanking regions were found. First, a cross-species amplification test was performed in 24 selected primer pairs (Table 1) using the Qiagen Multiplex PCR plus Kit (Qiagen, Hilden, Germany) with the 5′-tailed primer method (Boutin-Ganache et al. 2001) with CAG-tailing (5′-CAGTCGGGCGTCATCA-3′) and the PIG tailing (5′-GTTT-3′) method (Brownstein et al. 1996) following Takayama et al. (2011). We applied single-plex PCR in touchdown thermal cycling programs as follows: initial denaturation at 95 °C for 5 min, followed by first 15 cycles of denaturation at 95 °C for 30 s, annealing at 63 °C for 90 s (decreased 0.5 °C per cycle), and extension at 72 °C for 60 s; and by second 25 cycles of denaturation at 95 °C for 30 s, annealing at 55 °C for 90 s, and extension at 72 °C for 60 s; a final extension step was performed at 60 °C for 30 min. An automated sequencer (ABI 3130xl, Applied Biosystems, CA, USA) and GeneMarker (SoftGenetics LLC, PA, USA) were used for scoring of the PCR products.

**Table 1:** Characteristics of 24 microsatellite markers developed in *Robinsonia masafuerae*

Locus	Accession No.	Primer sequences (5′-3′)^a^	Repeat motif	*N*_A_	Length of fragments (bp)^b^					
					*R. evenia*	*R. gayana*	*R. gracilis*	*R. masafuerae*	*R. saxatilis*	*R. thurifera*
RM-HD5GH*	AB739985	F: [cag]TGGACAAATAATATGGGACGG	(AT)_7_	2	123	123	123	123/125	123	123
		R: [pig]TGTGTGTCTAGTGGTCCACATTC								
RM-G3THW*	AB739986	F: [cag]AATGGAATTCCCATCATCAAA	(GAA)_8_	5	190/193	187	181/184	184/187	193	187/190
		R: [pig]TGAAGAATACCGGTTGAAGC								
RM-HCT78*	AB739987	F: [cag]TCAAGCTGCTCAAAGAAAGG	(ATA)_11_	5	213/216	210/213	216	231	213/216	210/225
		R: [pig]TGGCCAAACTATGAAGTCCA								
RM-HIFPY*	AB739988	F: [cag]CGGTTAGCTCATCTCGGTTC	(AC)_8_	5	126	120	120	122	120/128	120/134
		R: [pig]GTGTTGTGGTAGCGATGGTG								
RM-HBJ9H*	AB739989	F: [cag]TTCAATTCCCTTTAGAGTTGGG	(AT)_7_	3	202	194	194	202	192	192
		R: [pig]GAATTCGTCTCTTTAGGTATCGG								
RM-HJIWZ*	AB739990	F: [cag]ATCCGGGTTTCCATATGTCC	(TG)_6_	2	259	259	261	259	259/261	259
		R: [pig]GAGTGGGAAAGTGTTAGAAAGCA								
RM-HMDCO*	AB739991	F: [cag]AGCATCACTGGTGTCAACCC	(GT)_9_AT(GT)_3_AT(GT)_6_	10	128/130	134/140	122/144	126/136	160	140
		R: [pig]CACATTTCCGTGATAAGTTAAGAGC								
RM-HBZZR*	AB739992	F: [cag]GTAGATGAACCCGGCTGTG	(AT)_6_	6	195	189/203	195/201	195/197	201/207	195
		R: [pig]TCCGGCGACTACTCTTTAACA								
RM-HGNFW*	AB739993	F: [cag]CAACAAGGACATTTCAACATTCA	(GT)_7_	3	217	215	219	217	215	215
		R: [pig]AATAGGTTGACGAGATCAAGGG								
RM-HGG8M*	AB739994	F: [cag]AGTATGGCAAGTTGGGTTGC	(TG)_7_	5	290	294/296	286	290	288	288
		R: [pig]TGACTGTAAGTAATGTGCAAGATGA								
RM-G07PU	AB739995	F: [cag]TCCGTATCATCAGGGTGTGA	(TTA)_8_	3	129	126	126	126	123	129
		R: [pig]TTTCAAGATGTAACCATTCATCA								
RM-G7UI4	AB739996	F: [cag]CAAGCTATGACGTTGAGGCA	(GT)_11_	3	127	127	129	127/130	127	127
		R: [pig]TTGCATTATTTATATGGGTTGACG								
RM-GSNGV	AB739997	F: [cag]CATGTTCAACAAGATCAACAACA	(CAA)_8_	4	129	123	123	129/135	126	126
		R: [pig]GTGTACAATTGGTGGAATGGG								
RM-HH90V	AB739998	F: [cag]CAAAGTGGGTACCTAAATTGCAC	(TG)_7_	2	165/167	165/167	165/167	165	165/167	165/167
		R: [pig]TTTCAAAGTGTACCGTTCCC								
RM-CO292	AB739999	F: [cag]AGAATTACACAGCCCTGCCA	(AT)_7_	1	205	205	205	205	205	205
		R: [pig]GAAGCTCCCATCGAAATTCA								
RM-HE3XN	AB740000	F: [cag]AAGAGAGGGTGAGATTGTGTCA	(TC)_9_	7	119/121	133/137	119/135	117/121	×	121
		R: [pig]TTTACACAACAAACAGACACCC								
RM-CO472	AB740001	F: [cag]ACATGAAGCGGTTATGGGAG	(CA)_9_	3	133	133	×	127	133/135	135
		R: [pig]AGGCAGAAACAATAATCCGC								
RM-G05Q6	AB740002	F: [cag]AAACAGAGGCAATGGTACGTG	(TA)_6_	2	277	279	279	277	×	×
		R: [pig]AGAATGTTACAATGGACCTCCTC								
RM-G1J9T	AB740003	F: [cag]CCTTGCGAGAGGTCAATTTC	(TAA)_8_	2	133	×	×	133/142	133	133
		R: [pig]CAGATGATCTTGAATCGGTTATATG								
RM-HA7DJ	AB740004	F: [cag]ACATGTCCCTTGTTGGTCTTAG	(TA)_6_	3	252	260	×	258	×	×
		R: [pig]TTGTTACATTCTCATGCACTTGG								
RM-CO442	AB740005	F: [cag]TTCAGATGGGCAAATTACACC	(TA)_11_	1	×	178	176	×	×	×
		R: [pig]TTCATTTGTCACCTGATCCTTC								
RM-HKUSB	AB740006	F: [cag]TTCTTCTCCGGTTGATTTCG	(AC)_9_	1	×	×	×	×	221	221
		R: [pig]TTCCTTATCCTCTTCTGTGTTCA								
RM-G1L4T	AB740007	F: [cag]CTCTTCGCATCTGGCATTTA	(AT)_10_	2	×	×	×	124/126	×	×
		R: [pig]GGAACGGACGCGTATATTGA								
RM-G5RN7	AB740008	F: [cag]AACTTCGGTCCCATTGTCAC	(GT)_8_GA(GT)_9_	0	×	×	×	×	×	×
		R: [pig]TGTTATCCGCTACAACTTCTTTGA								

*N*_A_, total number of alleles in the six species; *, ten microsatellite markers selected for the polymorphic test in Table 2; ×, amplification unsuccessful

^a^[cag], CAG tailing (5′-CAGTCGGGCGTCATCA-3′); [pig], GTTT tailing (5′-GTTT-3′)

^b^Length of fragments is shown by a single number (homozygote) or by two numbers separated by a slash (heterozygote)

Next, we selected the ten best of the 24 markers, and confirmed the polymorphism using multiple individuals of three populations, one from each of the three species (Table 2). We applied multi-plex PCR as follows: RM-HD5GH, RM-G3THW, RM-HCT78 with 6-FAM, RM-HIFPY, RM-HBJ9H, RM-HJIWZ with VIC, RM-HMDCO, RM-HBZZR with NED, RM-HGNFW, RM-HGC8M with PET. Two to 17 alleles per locus were detected in the three populations, and expected heterozygosity ranged from 0.000 to 0.847 (Table 2). A significant heterozygote deficiency was detected for zero to two markers in each population, and no significant linkage disequilibrium between markers was observed in both populations (*P* < 0.05, after Bonferroni correction) using GENEPOP 4.0 (Raymond and Rousset 1995). Ten microsatellite markers present easily scorable polymorphic peaks in the six species of *Robinsonia*, rendering these markers useful for populational genetic studies.

**Table 2:** Results of ten microsatellite markers in *R. evenia*, *R.* *gayana*, and *R.* *gracilis*

Marker	All	*R. evenia* (n = 37)				*R. gayana,* population 2 (n = 18)				*R. gracilis*, population 2 (n = 29)			
	*T*_A_	*N*_A_	*H*_O_	*H*_E_	*F*_IS_	*N*_A_	*H*_O_	*H*_E_	*F*_IS_	*N*_A_	*H*_O_	*H*_E_	*F*_IS_
RM-HD5GH	4	1	0.000	0.000	NA	1	0.000	0.000	NA	4	0.241	0.640	0.623*
RM-G3THW	5	2	0.351	0.407	0.136	3	0.056	0.156	0.644*	2	0.483	0.485	0.005
RM-HCT78	10	6	0.595	0.589	−0.009	8	0.833	0.844	0.013	3	0.552	0.527	−0.046
RM-HIFPY	8	4	0.541	0.551	0.019	4	0.500	0.608	0.178	3	0.138	0.347	0.603*
RM-HBJ9H	5	3	0.270	0.349	0.226	2	0.278	0.313	0.113	3	0.379	0.511	0.258
RM-HJIWZ	2	1	0.000	0.000	NA	1	0.000	0.000	NA	1	0.000	0.000	NA
RM-HMDCO	17	5	0.432	0.581	0.255	10	0.944	0.847	−0.115	3	0.448	0.424	−0.058
RM-HBZZR	5	1	0.000	0.000	NA	5	0.222	0.684	0.675*	2	0.552	0.471	−0.172
RM-HGNFW	3	2	0.054	0.053	−0.028	1	0.000	0.000	NA	3	0.103	0.099	−0.042
RM-HGC8M	5	1	0.000	0.000	NA	3	0.778	0.549	−0.416	1	0.000	0.000	NA

*T*_A_, total number of alleles in the three populations; *N*_A_, total number of alleles within each population; *H*_O_, observed heterozygosity; *H*_E_, expected heterozygosity; *F*_IS_, inbreeding coefficient; *NA*, not applicable

*departs significantly from HWE (*P* < 0.05)
